# An Improved Microaneurysms Detection for Diabetic Retinopathy Screening Using YOLO

**DOI:** 10.3390/biomedicines14020359

**Published:** 2026-02-04

**Authors:** Sarni Suhaila Rahim, Ankur Deo, Rafia Mumtaz, Vasile Palade

**Affiliations:** 1 Centre for Computational Science and Mathematical Modelling, Coventry University, Innovation Village 10, Coventry CV1 2TL, UK; deoa2@uni.coventry.ac.uk (A.D.); ab5839@coventry.ac.uk (V.P.); 2 Centre for Advanced Computing Technology (C-ACT), Fakulti Teknologi Maklumat dan Komunikasi, Universiti Teknikal Malaysia Melaka (UTeM), Hang Tuah Jaya, Durian Tunggal, Melaka 76100, Malaysia; 3 KPIT Technologies Ltd., 16 Av De La Grande Armee, 75017 Paris, France; 4 School of Electrical Engineering and Computer Science (SEECS), National University of Sciences and Technology (NUST), Sector H-12, Islamabad 44000, Pakistan; rafia.mumtaz@seecs.edu.pk

**Keywords:** diabetic retinopathy, microaneurysms detection, YOLO, retinal fundus imaging, deep learning

## Abstract

**Background/Objectives**: Diabetic retinopathy (DR) is a chronic, progressive complication of diabetes mellitus and remains one of the leading causes of vision impairment worldwide, particularly when early pathological changes go undetected or untreated. The earliest clinically identifiable biomarkers are microaneurysms, which are minute, round dilatations of capillary walls. Retinal abnormalities of a broad spectrum are indicative of the condition. This paper introduces a novel automated screening system for DR that prioritises the detection of these early indicators. **Methods**: The proposed approach integrates advanced image processing techniques based on the circular Hough transform and the YOLOv9 model, to localise and detect microaneurysms in colour fundus images. **Results**: Several system prototype versions were developed and evaluated. The final, best-performing YOLOv9-based model achieved an accuracy of 91%, representing a substantial performance improvement compared with the circular Hough transform. **Conclusions**: The developed models effectively address the issue of significant image processing challenges in lesion detection as well as small and class imbalance data, which are recurring constraints in medical image analysis.

## 1. Introduction

Diabetic retinopathy (DR) is a significant microvascular consequence of diabetes mellitus and a primary cause of preventable blindness worldwide. In its initial phases, DR is typically asymptomatic, leaving patients often oblivious to both their diabetic condition and the retinal pathological alterations. Therefore, effective screening programmes are critical for the timely identification of DR and for identifying patients at elevated risk of vision loss.

Diabetic retinopathy progresses through a well-characterised spectrum of microvascular pathology. Visual symptoms, such as reduced acuity, metamorphopsia, and vision loss, may arise at any stage. Early DR is typically asymptomatic, yet fundoscopic or imaging modalities may detect subtle retinal changes. The earliest manifestations include microaneurysms, followed by the emergence of cotton wool spots, intraretinal haemorrhages, and microvascular abnormalities. With progression to proliferative DR, pathological neovascularisation occurs, characterised by the growth of fragile, permeable vessels predisposed to rupture. This process significantly increases the risk of vitreous haemorrhage, fibrovascular proliferation, and tractional retinal detachment, often resulting in irreversible visual impairment [1].

**Figure 1:**
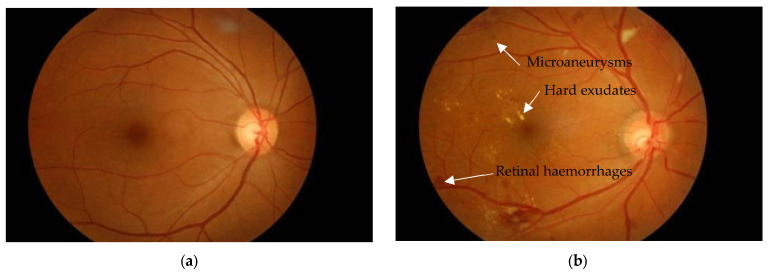
Diabetic retinopathy signs representation. (**a**) Normal retina [2]; (**b**) Features of diabetic retinopathy in fundus photography [2].

This paper concentrates on the detection of microaneurysms, one of the earliest and most clinically significant indicators of diabetic retinopathy. Microaneurysms appear as small red dots on the retina, and result from the focal dilatation of weakened capillary walls. Figure 1 shows a visualisation of diabetic retinopathy signs.

There are various ways to analyse the fundus, but most of them still have troubles with the way retinal images change in the actual world, for example, when the illumination is uneven the contrast is low, or the anatomical forms may not be perfect. Old methods like the circular Hough transform depend on rigid geometric rules, and even older versions of YOLO had troubles in capturing small retinal patterns. To fill this gap, we present a YOLOv9-based method that is more able to deal with these differences. We are motivated by the fact that YOLOv9’s stronger feature extraction and anchor-free detection can better capture complicated retinal patterns, especially in populations of various ethnical background. This study’s key contributions are: (1) proposing a YOLOv9-based solution that is better suited to these problems; and (2) showing that the proposed solution works better than other methods on the available eye fundus image datasets.

The YOLOv9 architecture was chosen due to its superior suitability for medical image analysis, especially in the detection of subtle, low-contrast features like microaneurysms. Enhanced feature fusion improves the detection of subtle details, and heightened noise robustness increases reliability across diverse retinal image quality conditions.

This paper is organised as follows. Section 2 presents a detailed analysis of the state of the art on automated DR detection, with a focus on microaneurysm detection using YOLO and other deep learning techniques. The proposed framework for microaneurysm detection in retinal fundus images is presented in Section 3, which combines classical image processing techniques with effective deep learning architectures. Section 4 presents the obtained experimental results of the proposed system variants. Meanwhile, Section 5 discussed the main findings and their implications, and also suggests potential directions for future research. Lastly, Section 6 provides a concise summary of the primary contributions of this work.

## 2. Related Work

Numerous systems have been proposed in the literature for the automated detection and diagnosis of diabetic retinopathy. Some of these systems provide general classification, distinguishing retinal fundus images based on the presence or absence of retinopathy [3,4,5,6,7,8]. Others are designed to detect specific pathological features associated with the condition, such as microaneurysms, haemorrhages, exudates, and related abnormalities [9,10,11,12,13,14,15,16,17,18,19,20,21,22,23,24,25,26,27,28,29,30]. Among these, the automatic localisation of microaneurysms remains particularly challenging due to their small size and subtle presentation [17,18,19,20,21,22,23,24,25,26,27,28,29,30,31], necessitating further research to develop more robust and accurate detection methods.

A variety of systems for microaneurysm detection have been proposed in the literature, employing diverse methodological approaches to enhance diagnostic reliability. Despite these efforts, microaneurysm detection remains a technically demanding task. As Rahim et al. [31] observe, accurate quantification is impeded by the typically high lesion density, frequent spatial overlap, and the morphological similarity of microaneurysms to blot haemorrhages, all of which complicate their differentiation in retinal fundus images.

Deep learning has been widely adopted in the automated screening of diabetic retinopathy using retinal fundus images. Studies have introduced various deep learning-based frameworks for DR detection, utilising diverse neural network architectures, datasets, training strategies, and grading protocols, yielding varying levels of diagnostic performance. In contrast, the works reported in [9,10,11,12,13,14,15,16,17,18,19,20,21,22,23,24,25,26,27,28,29] focus specifically on the detection of microaneurysms, an early hallmark of DR, through the integration of deep learning-based classification models, aimed at achieving precise and reliable localisation of these lesions.

In our earlier work, we introduced a novel automated screening system for diabetic retinopathy, designed to detect the earliest clinically indicators of the disease, namely microaneurysms [30]. The system integrates several feature extraction techniques in combination with the circular Hough transform for microaneurysm detection. In addition, fuzzy histogram equalisation was employed during the preprocessing stage of the retinal fundus images, which demonstrably enhanced the accuracy of microaneurysm identification. Next, we investigated a hybrid framework that integrates fuzzy image processing techniques with deep learning models for the automated detection of microaneurysms in colour fundus images. The study aimed to enhance both the accuracy and efficiency of microaneurysms detection, as microaneurysms represent one of the earliest clinical indicators of diabetic retinopathy—a leading cause of visual impairment globally. The findings demonstrated the potential of the proposed approach to outperform conventional methods and certain deep learning-based techniques, particularly in terms of computational efficiency and resource utilisation [31].

The detection of microaneurysms in diabetic retinopathy screening is hindered by substantial class imbalance, as pathological lesions constitute only a minor fraction of the overall retinal data relative to non-pathological regions [17,18]. Recent deep learning approaches have addressed this issue through advanced data augmentation techniques, optimised loss functions, and refined architectural innovations, thereby improving detection accuracy while retaining clinical relevance [19,20]. Evidence increasingly supports the efficacy of integrative strategies; combining generative adversarial networks, ensemble learning, and attention-based mechanisms, in overcoming class imbalance and enhancing the robustness and generalisability of microaneurysm detection in real-world screening environments [21,22].

The Synthetic Minority Oversampling Technique (SMOTE) is a pivotal tool for addressing class imbalance in microaneurysm detection, with advanced variations regularly surpassing conventional data-level resampling techniques [23]. Generational Adversarial Networks (GANs) have substantially enhanced the production of high-quality artificial retinal images with adjustable disease characteristics, thereby enabling data augmentation for the identification of unbalanced microaneurysms [24,25]. DenseNet-121 models, when trained on the imbalanced APTOS 2019 dataset and enhanced with suitable class balancing algorithms, have attained an accuracy of 97.30%, significantly above the performance of hybrid VGG16-XGBoost models, which obtain an accuracy of 79.50% [17]. The integration of extensive preprocessing pipelines utilising Ben Graham’s enhancement techniques and modern data augmentation methods has allowed detection systems to attain accuracy rates of 99.04%, accompanied by a quadratic weighted kappa score of 0.922 [26].

Inference was completed within 188 s in [27], and advanced convolutional neural network (CNN) architectures that utilise SqueezeNet, in conjunction with targeted data augmentation strategies, have exhibited performance that exceeds 85% accuracy, 89% specificity, and 80% sensitivity on validation cohorts consisting of 5000 retinal images. Furthermore, the integration of sophisticated preprocessing techniques and background removal has been demonstrated to substantially enhance model performance, with reported increases in validation accuracy of up to 90.60% when deployed on class-imbalanced datasets [28]. Advanced preprocessing techniques, such as data augmentation, image resizing, and contrast-limited adaptive histogram equalisation (CLAHE), are necessary to ensure consistent model performance across heterogeneous imaging conditions and variations in acquisition equipment [29].

The You Only Look Once (YOLO) algorithm, introduced by Redmon et al. [32], reformulates object detection as a single regression task that simultaneously predicts bounding boxes and class probabilities for each grid cell in one forward pass of a convolutional network. Its unified architecture enables end-to-end optimisation of detection performance and runs in real time. Table 1 summarises the development, architecture, strengths and limitations of each YOLO version.

The extraordinary speed, precision, and accessibility of YOLO models have made them highly influential in the field of computer vision [33]. The real-time detection capabilities make them well suited for fast-changing scenarios such as live video monitoring and automated tracking. These open-source models have the potential to foster extensive collaboration within the research community, drive innovation, and enable engineers to develop efficient, high-performance visual recognition systems. They achieve state-of-the-art accuracy, particularly in versions like YOLOv7 [33].

Zhang et al. [34] recently conducted a study on a multimodal AI framework for the automated generation of ophthalmic ultrasonography reports. This framework integrates image and text features through three modules: YOLOv11s-based object detection, YOLOv11s-PaddleOCR text extraction, and an enhanced VanillaNet disease classifier. The method achieves high accuracy in all of its tasks, thereby improving diagnostic efficiency and alleviating the scarcity of trained ophthalmologists.

Ardelean et al. [35] proposed a study that evaluates the performance of sophisticated object detection models, such as YOLOv8–v12, YOLO-World, YOLOE, and RT-DETR, in the detection of retinal pathologies in OCT images. The results indicate that YOLOv12 obtains the optimal balance of accuracy and efficiency, while YOLOE provides the highest overall performance, thereby establishing a robust foundation for automated OCT-based diagnosis.

Veena and others [36] presented FFA-Lens, a web-based application utilising the YOLOv8 architecture for automated detection of 25 common lesions in fundus fluorescein angiography images. With precision and recall values exceeding 0.8, the system demonstrates strong potential to improve diagnostic efficiency and support early detection in the management of chronic ocular diseases.

Rizzieri et al. [37] proposed a YOLOv8-based AI model for early identification of myopia from fundus images, with 85% accuracy with robust precision and recall. The method shows that AI-based screening could help in early detection and prevention of myopia. Hussein and Al-Saadi [38] came up with a YOLO-based deep learning method for accurately identifying and separating the optic disc in retinal fundus photos. The method is better than others since it has a recall rate of 100%, an accuracy rate of 99.5%, and a precision rate of 99.9%. This is a good place to start when it comes to automatically diagnosing retinal problems.

Research utilising several YOLO-based algorithms for the detection and classification of diabetic retinopathy demonstrates significant improvements in accuracy, speed, and practical applicability. Sait [3] developed a lightweight YOLOv7-based DR detection model utilising MobileNet V3, attaining 98.4% accuracy on the APTOS and EyePacs datasets, appropriate for resource-constrained environments. Akella and Kumar [4] employed YOLOv3 to classify DR across five stages from fundus images, achieving high precision, sensitivity, and mAP, outperforming existing methods. Moya-Albor et al. [5] combined YOLOv8 with a bio-inspired watermarking method for secure fundus image analysis, preserving DR grading accuracy while ensuring image authentication. Ramesh et al. [6] built a special YOLOv5 AI toolkit to find DR and its clinical symptoms in high-resolution fundus images, which was 91% accurate and 100% specific. Lalitha and Padyana [7] introduced a YOLO-RF hybrid model for multi-stage DR detection, attaining 99.3% accuracy, 97.2% precision, and 99.1% recall on the Kaggle and IDRiD datasets. Kumar and Dhanalakshmi [8] introduced EYE-YOLO, an improved Tiny YOLOv7 model that incorporates Focal-EIOU loss and multi-spatial pyramid pooling. This model has the potential to achieve a 30.81% increase in mAP for eye diseases, including DR.

Researchers are aiding the automated identification of microaneurysms in diabetic retinopathy with YOLO-based models, enhancing early diagnosis and clinical management. Zhang et al. [39] introduced MA-YOLO, an improved YOLOv8 model integrated with SwinIR super-resolution and Wise-IoU loss, with exceptional precision (97.98%), recall (88.23%), F1 score (92.85%), and average precision (94.62%) for microaneurysm detection in FFA pictures, surpassing other leading models. Santos et al. [40] developed a YOLOv5-based methodology that incorporates image processing, data augmentation, and transfer learning for the detection of fundus lesions, including microaneurysms. This strategy yielded enhanced F1 scores and mean Average Precision (mAP) on the DDR [41] and IDRiD [42] datasets in comparison to previous studies. Alyoubi et al. [43] implemented a hybrid framework combining a CNN classifier with an enhanced YOLOv3 model to detect and localise diabetic retinopathy lesions, including microaneurysms, in fundus images, achieving 0.216 mAP for lesion detection and an overall accuracy of 89%, surpassing existing diagnostic methods. Akut [44] developed a YOLO-based object detection model that simultaneously detects and localises microaneurysms on the retina, allowing ophthalmologists to identify their precise location efficiently and reducing the need for manual intervention.

It may be concluded that a range of approaches and deep learning classifiers have been proposed for the detection of microaneurysms. The YOLO strategies that have been employed in the area of diabetic retinopathy screening and analysis, including approaches to detect microaneurysms, are summarised in Table 2. Nevertheless, the automated detection of microaneurysms remains a significant challenge, and further research is needed to develop more robust and reliable techniques capable of accurately identifying these early indicators of diabetic retinopathy. This work presents a modified YOLO variation designed for the identification of microaneurysms, which are among the most challenging lesions to diagnose in diabetic retinopathy, acknowledging the strengths of YOLO models in speed and precision.

**Table 1:** Summary of YOLO version performances [45,46].

Version	Proposed Year	Architecture/ Key Features	Performance/Strength	Limitations
YOLOv1	2015	Divides image into an *S* × *S* grid; single-stage detection with 24 convolutional and 2 fully connected layers; trained on ImageNet	Real-time detection capability with simple end-to-end training	Limited accuracy for small and overlapping objects; coarse localisation
YOLOv2	2016/2017	Introduced anchor boxes, batch normalisation, residual networks, and fine-tuning with 448 × 448 images	Improved accuracy and speed; better handling of multiple objects	Still struggles with small-scale objects and complex backgrounds
YOLOv3	2018	Based on Darknet-53; employs multi-scale detection at three levels using nine anchor boxes	Higher precision and robustness; better detection across object sizes	Computationally heavier; slower than lightweight successors
YOLOv4	2020	Uses CSPDarknet53 backbone, spatial pyramid pooling, and cross-stage partial connections	Achieved 43.5% AP on MS COCO; real-time detection at 62 FPS (608 × 608)	Complex training pipeline; large model size
YOLOv5	2020	Lightweight PyTorch-based model; supports GIoU loss and binary cross-entropy for class probability	Fast inference, highly customisable, and efficient for deployment	Proprietary at first; lacks official paper and transparency issues
YOLOv6	2022	Introduced EfficientRep Backbone, Rep-PAN Neck, and decoupled head; anchor-free training with SimOTA and SIoU loss	Hardware-optimised; enhanced accuracy-speed balance	Less effective for dense object scenarios
YOLOv7	2022	Incorporates E-ELAN for efficient gradient flow; optimised for memory and computational efficiency	Strong performance with reduced training time; supports Roboflow dataset integration	Slightly slower than YOLOv8; limited scalability for very large datasets
YOLOv8	2023	Anchor-free design; scale-aware training with mosaic augmentation; refined loss (VFL + DFL + CIOU)	Excellent generalisation and multi-class detection; developer-friendly	High memory demand during training; reduced interpretability
YOLOv9	2024	Programmable Gradient Information framework; enhanced feature extraction and transformer integration	Outperforms YOLOv5-v8 on MS COCO with higher mAP	Increased architectural complexity; training resource-intensive
YOLOv10	2024	Hardware-efficient design with fewer parameters; real-time edge AI optimisation	Lower latency and smaller model size; 46% less latency vs. YOLOv9-C	Limited open-source ecosystem; trade-off between compression and accuracy
YOLOv11	2024	Multi-task architecture for detection, segmentation, classification, keypoints, and OBB; modular scaling from YOLOv11n to YOLOv11x	Achieves up to 54.7% mAP on COCO; strong multi-domain capability	Large variants are computationally demanding
YOLOv12	2025	Attention-centric real-time architecture; refined backbone for contextual feature learning	Higher mAP and lower latency on COCO; state-of-the-art performance	Recently released-limited empirical validation beyond COCO

**Table 2:** YOLO-based methods for diabetic retinopathy detection summary.

Author [Ref.]	Type of Detection	YOLO Model Used	YOLO Performance
Sait [3]	Diabetic retinopathy (DR)	YOLOv7	Achieve 98.0% and 98.4% accuracy with F1-scores of 93.7 and 93.1 on APTOS and EyePACS datasets, respectively; reduced computational complexity with fewer FLOPs and parameters
Akella and Kumar [4]	DR	YOLOv3	Demonstrated high precision and sensitivity with superior accuracy and reduced implementation time compared with prior models
Moya-Albor et al. [5]	DR	YOLOv8	Maintained consistent DR grading accuracy on both original and watermarked fundus images, confirming model robustness and diagnostic reliability
Lalitha and Padyana [7]	DR	YOLO-RF (YOLO integrated with Random Forest)	Achieved 99.3% accuracy, precision of 97.2, and recall of 99.1 on Kaggle and IDRiD datasets; outperformed traditional machine learning classifiers
Alyoubi et al. [43]	DR and lesions	YOLOv3 (with CNN fusion)	Combined CNN512 and YOLOv3 achieved 89% accuracy, 89% sensitivity, and 97.3% specificity; outperformed existing DR detection systems
Kumar and Dhanalakshmi [8]	Cataract, glaucoma, retinal disease, normal	EYE-YOLO (enhanced Tiny YOLOv7)	Attained 30.81% higher mAP and up to 28% higher precision than Tiny YOLOv7; exceeded YOLOv5–v8 variants in all metrics
Ramesh et al. [6]	Microaneurysms, haemorrahages, exudates	YOLOv5	Accuracy improved from 79.5% to 91%, with sensitivity of 100%; effectively identified multiple lesion types using confocal true-colour fundus images
Zhang et al. [39]	Microaneurysms	MA-YOLO (YOLOv8 + SwinIR)	Achieved precision 97.98%, recall 88.23%, F1-score 92.85%, and AP 94.62%, surpassed YOLOv5, YOLOv7, YOLOX, SSD, and RetinaNet
Santos et al. [40]	Microaneurysms, exudates, haemorrhages	YOLOv5	Recorded mAP of 0.263 (validation) and 0.154 (test) on DDR dataset; demonstrated improved lesion detection compared with prior studies despite challenges in small-object recognition
Akut [44]	Microaneurysms	YOLO-based object detection model	Integrated microaneurysms detection and localisation in a single framework, automating both diagnostic stages for early DR identification

## 3. Materials and Methods

### 3.1. Materials

#### 3.1.1. Datasets

The experimental work conducted in this study employed three distinct datasets. The first dataset, The Retinopathy Online Challenge (ROC), showed different ways to find microaneurysms [47]. The competition dataset has 50 training photos and 50 testing images, each with a reference standard. The pictures are available in three different sizes: 1389 × 1383 pixels, 768 × 576 pixels, and 1058 × 1061 pixels. The findings of the competition demonstrated that finding microaneurysms is still a big problem for both automated systems and human experts.

The second dataset was the Indian Diabetic Retinopathy Image Dataset (IDRiD) [42]. This dataset is said to be representative of an Indian population. The collection contains 516 eye fundus photos that are stored in JPEG format and have a high definition of 4288 × 2848 pixels. The pictures were taken with a Kowa VX-10 alpha digital fundus camera with a 50-degree angle (Kowa Company Ltd., Tokyo, Japan). The camera was located at the Eye Clinic in Nanded, Maharashtra, India. The macula is the core of all the images. The collection contains expert annotations of common diabetic retinopathy indicators, together with details about the severity level of diabetic retinopathy and the existence of diabetic macular oedema for each image.

The third dataset was the DDR dataset, used to evaluate deep learning models and their clinical applicability in lesion recognition [41]. It contains 13,673 fundus images from 9598 patients, graded into six classes by seven independent reviewers based on image quality and diabetic retinopathy severity. A subset of 757 images includes annotations for four DR-related lesion types. The dataset has been used to test deep learning performance in image classification, semantic segmentation, and object detection.

From these datasets, a stratified subset of 800 images was selected based on the presence and number of microaneurysms to ensure a balanced distribution of diabetic retinopathy severity levels and lesion presence. The images were split into 70% training and 30% testing, maintaining the same clinical distribution across all splits. This approach allowed us to create a dataset that is both manageable for training and sufficiently representative of the variability present in the original collections. All selected images were rescaled to a uniform resolution of 768 × 576 pixels to allow consistent model training and evaluation.

#### 3.1.2. Implementation

All model implementations were carried out using the PyTorch 2.8.0 framework and the Python 3.14 programming environment, leveraging CUDA 12.3 for GPU acceleration on an NVIDIA RTX 4090, with a batch size of 32 and mixed-precision training (FP16).

### 3.2. Methods

#### 3.2.1. Image Pre-Processing

The pre-processing stage serves as the foundation of the proposed microaneurysm (MA) detection system, ensuring that each retinal image is carefully prepared for accurate and efficient analysis. The process begins by converting the original colour fundus image into a grayscale image, using an equal blend of the red and green channels (Gray = 0.5R + 0.5G). Although the green channel is commonly used in fundus image analysis due to its generally higher contrast for red lesions, preliminary experiments conducted on our dataset [30,31] showed that using the green channel alone resulted in a loss of local intensity variation around microaneurysms in darker or lower-exposure images. To mitigate this, we adopted a custom grayscale conversion (Gray = 0.5R + 0.5G), which preserves the high lesion contrast of the green channel while incorporating complementary vessel and background intensity cues from the red channel. This step simplifies the data while preserving the most diagnostically meaningful information, as these channels best capture retinal structures and lesion patterns. Next, median filtering is applied to remove unwanted noise such as uneven illumination or sensor artefacts, while still preserving delicate image details like fine vessels and small lesion spots. This balance between noise reduction and feature preservation is critical for reliable microaneurysm detection. The technique enhances the visibility of potential lesion regions by performing an image difference operation, wherein the median-filtered image is subtracted from the original greyscale image. This highlights subtle variations in intensity and contrast commonly associated with microaneurysms. The outcome is more refined, distinct, and informative image that establishes the foundation for accurate lesion identification in the following image processing phase.

#### 3.2.2. Image Processing

In the image processing phase, the primary objectives are to refine critical image features, isolate potential lesion areas, and identify candidate regions that may suggest the presence of microaneurysms. Initially, Contrast Limited Adaptive Histogram Equalisation (CLAHE) is applied to the pre-processed image to improve local contrast and reveal subtle pathological patterns. CLAHE enhance the visibility of microaneurysms while preventing the over-amplification of noise or bright artefacts, particularly in low-contrast retinal regions. Following this enhancement, adaptive thresholding is utilised to separate potential lesion areas from background, effectively binarising the image based on local intensity variations. To enhance detection accuracy, anatomical structures such as optic disc, blood vessels, hard exudates, and peripheral regions are excluded through a combination of region masking and threshold-based suppression to prevent the model from misidentifying these areas as lesions. Once these non-relevant regions are masked out, the refined image is analysed using the You Only Look Once version 9 (YOLOv9) deep learning model to accurately detect and localise microaneurysms. YOLOv9 employs convolutional neural networks to identify and localise microaneurysms with high spatial precision by analysing texture, shape, and contextual features within the image. The model generates bounding boxes around detected lesions, each with a confidence score, ensuring a reliable and accurate detection framework. In this work, YOLOv9 is employed due to the characteristics, such as speed, higher accuracy, computational efficiency, and more accurate results, making it a strong solution for detection of microaneurysms.

#### 3.2.3. Post-Processing

During the post-processing phase, the system meticulously examines and refines the identified regions to ensure the results are precise and applicable for clinical interpretation. The process begins by eliminating false detections, assessing the size and form of each region to exudate those likely attributable to noise, small vessel pieces, or distortions in the image. This ensures that only regions resembling real microaneurysms are kept. The refined detections are then turned into a binary mask that clearly marks the lesion areas, allowing the system to automatically count the number of microaneurysms, a key indicator of how advanced diabetic retinopathy may be. Ultimately, these identified regions are superimposed into the original retinal image, providing clinicians with a definitive visual confirmation of the lesions’ locations. This phase enables more straightforward interpretation of results for physicians, while bolstering confidence in the system’s precision, so offering a transparent and reliable tool for the early diagnosis and efficient screening of diabetic retinopathy.

#### 3.2.4. Training

The training phase is crucial in the development of our microaneurysm detection technology, enabling the model to reliably identify and localise even the minutest retinal lesions. This study employed the YOLOv9 architecture, refined via transfer learning, allowing the network to leverage pre-existing visual knowledge and adapt it to the specific patterns seen in retinal fundus images. The primary augmentation used was the flip-flop technique, consisting of random horizontal flips and random vertical flips, to make the model stronger and less likely to overfit. The network sees the same retinal structures from many different angles by flipping images horizontally and vertically. This makes the training data less similar. The model was trained using a meticulously tuned hyperparameter suite—an adaptive learning-rate scheduler, a batch size of 16, AdamW optimizer with weight decay, mixed-precision (FP16) training, and epoch-wise validation checkpoints to track loss and guard against performance regression. This regimen enables YOLOv9 to detect microaneurysms with high precision, supporting earlier and more reliable diabetic-retinopathy screening.

**Figure 2:**
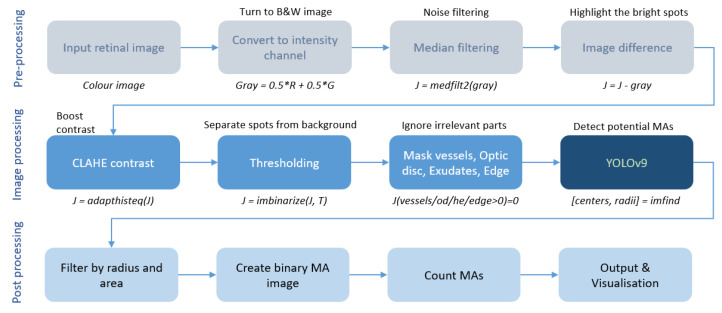
Flow diagram of the proposed systems.

Figure 2 shows the flow diagram of the whole work development.

**Figure 3:**
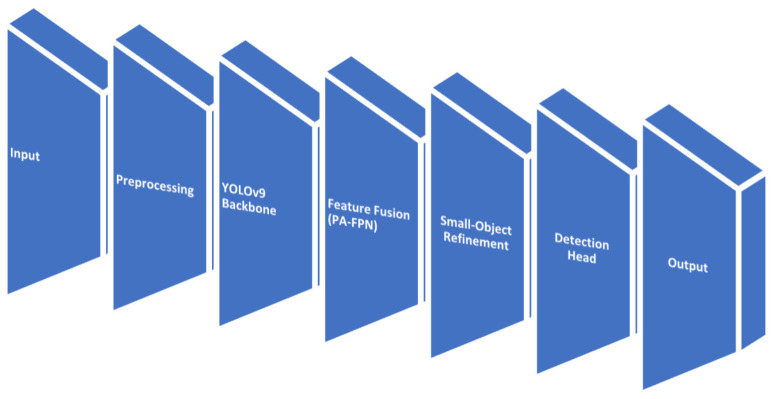
Basic YOLOv9 pipeline flowchart.

Figure 3 shows the basic pipeline diagram illustrating the YOLOv9 architecture within the proposed microaneurysm detection system.

**Figure 4:**
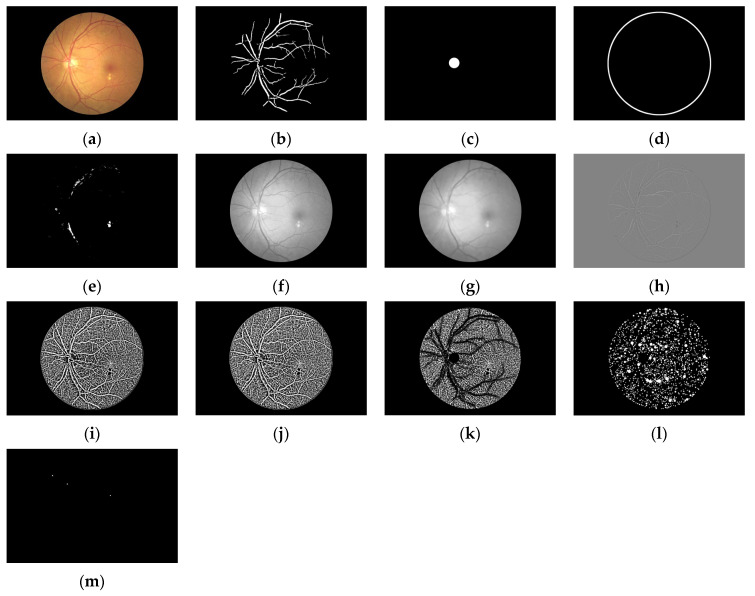
Processed images: (**a**) original colour image; (**b**) blood vessels detection; (**c**) optic disc detection; (**d**) edge detection; (**e**) hard exudates detection; (**f**) grayscale conversion; (**g**) median filtering, (**h**) image difference; (**i**) CLAHE; (**j**) adaptive thresholding; (**k**) masking; (**l**) YOLOv9 masking; (**m**) robust masking.

Examples of processed images output are shown in Figure 4. The preprocessed images include the blood vessels detection, optic disc detection, edge detection and hard exudates detection, grayscale conversion, median filtering, image difference, histogram equalisation, adaptive thresholding and masking.

## 4. Results

**Figure 5:**
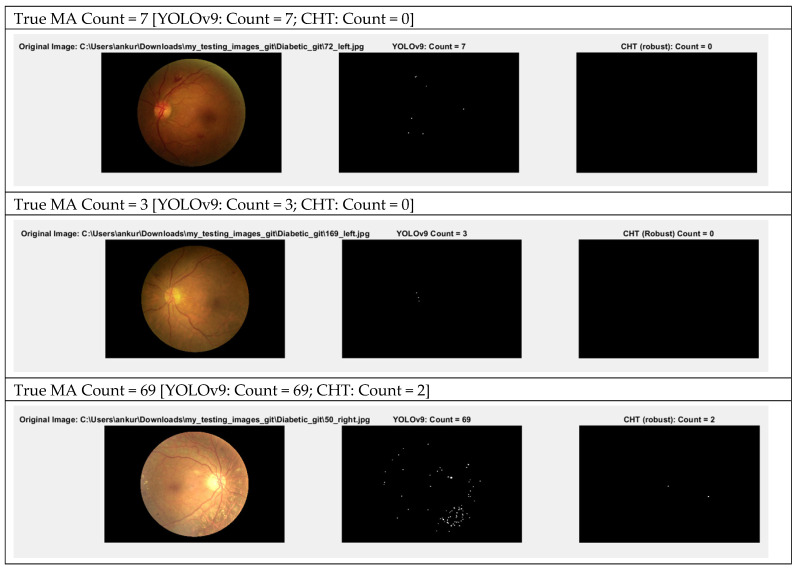
Comparison between output images between obtained by YOLOv9-based and CHT-based systems.

**Figure 6:**
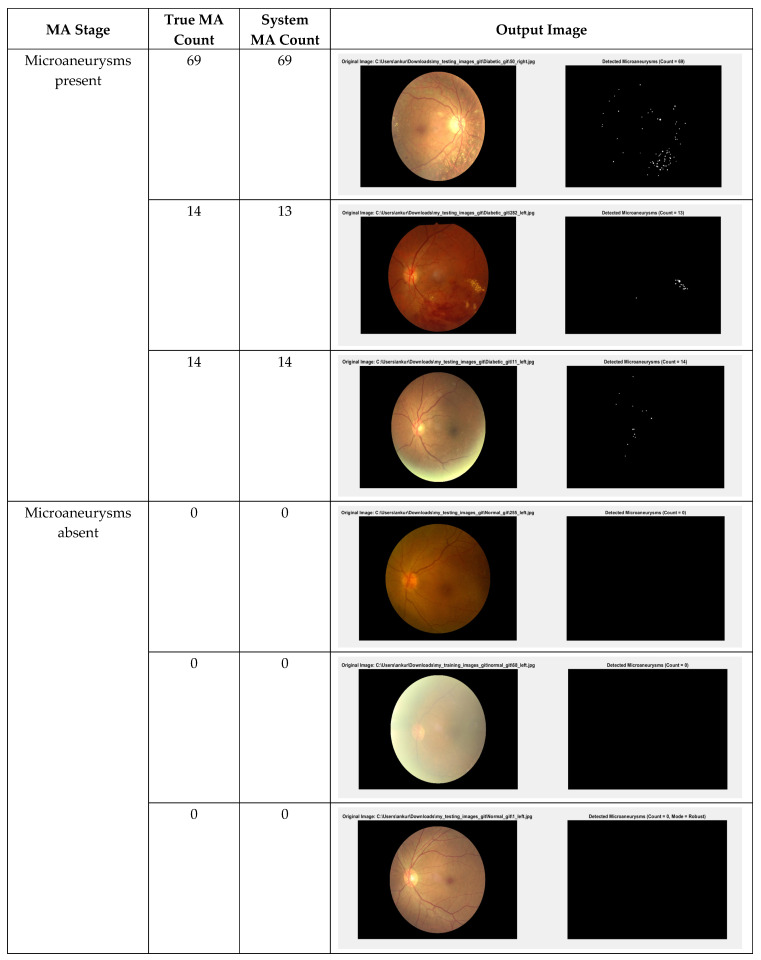
YOLOv9-based model performance for the microaneurysms detection on DDR and IDRiD datasets.

Figure 5 presents the output images generated from both systems. The first image column represents the original image, while the second and third image columns represent the comparison output image of microaneurysms (MA) detection between the YOLOv9-based framework and the Circular Hough Transform (CHT)-based framework, respectively. The output images of YOLOv9 capabilities in the detection of the microaneurysms from this study are presented in Figure 6.

Table 3 summarises the overall performance results of the two system variants. The performance analysis shows that the two proposed detection systems are very different from each other. System I, based on the Circular Hough Transform (CHT), relies on geometry-based detection and preprocessed binary candidate maps. System II, on the other hand, uses the YOLOv9 architecture and raw or preprocessed retinal pictures for CNN-based object recognition.

In terms of image processing metrics, the YOLOv9-based system markedly outperforms the CHT-based pipeline. System II demonstrates strong performance, with sensitivity (recall) ranging from 0.83 to 0.88 and specificity (precision) between 0.82 and 0.87. Its F1-scores are similarly robust, ranging from 0.82 to 0.87. In contrast, the CHT-based system performs noticeably lower, with sensitivity of 0.65–0.72, specificity of 0.60–0.68, and F1-scores between 0.62 and 0.70. Accuracy across 200 test images further emphasises the superiority of YOLOv9, registering 91% versus 56% for the CHT-based approach under a 5% tolerance.

From an infrastructural perspective, the YOLOv9 pipeline demonstrates substantially faster processing, handling each tile in approximately 50–200 miliseconds on a GPU, whereas the CHT-based system requires 2–5 s per image on a CPU. Furthermore, YOLOv9 effectively detects small, irregular lesions down to 3–5 pixels, whereas the CHT method is limited to circular blobs with radii of 5–25 pixels. Post-processing demands are also lower in the YOLOv9 system, with moderate operations such as confidence filtering and non-maximum suppression, in contrast to the high computational load associated with masking, thresholding, and radius filtering in the CHT pipeline. The YOLOv9-based system exhibits enhanced detection performance, computational efficiency, and adaptation to diverse lesion morphology, positioning it as a more effective method for early microaneurysm identification in retinal imaging.

Table 4 presents some examples of the image detection performance of both proposed system variants in this study. A comparison of the two system variants shows that System II (YOLOv9) generally detects microaneurysms more accurately than System 1 (CHT). For examples, in images with higher MA counts such as 45_right [MA true count = 63] and 58_right [MA true count = 48], System II closely matches the true counts (62 and 48, respectively), whereas System I underestimates them (55 and 42). Similarly, for images with diminished MA counts, such as 53_left [MA true count = 12] and 59_left [MA true count = 5], System II accurately determines the precise counts, while System I demonstrates slight overestimations or underestimations. System II exhibits superior consistency in detection performance across various lesion densities as compared to the CHT-based method.

## 5. Discussion

Comparing the results of this study to the traditional Circular Hough Transform (CHT) method, it is evident that a YOLOv9-based deep learning methodology enhances microaneurysm (MA) detection for diabetic retinopathy screening. Across various performance parameters, YOLOv9 consistently outperformed the geometry-driven CHT pipeline. Sensitivity and specificity were both excellent, generally above 0.8, and F1-scores indicated similar robustness, whereas the CHT-based system routinely performed poorly. Accuracy over 200 test images further highlighted this difference: YOLOv9 correctly identified MAs with 91% accuracy compared with 56% for the CHT approach under a 5% tolerance threshold. These findings suggest that the deep learning system is not only more reliable in localising MAs but also more capable of managing the wide variability in lesion morphology and density that is characteristic of retinal images.

From a practical standpoint, YOLOv9 offers additional advantages beyond detection accuracy. The system processes each image tile in 50–200 milliseconds on a GPU, in contrast to the 2–5 s per image required by the CHT pipeline on a CPU, indicating a clear advantage for high-throughput or real-time screening applications. Moreover, YOLOv9 can detect small and irregularly shaped lesions as small as 3–5 pixels, whereas the CHT approach is limited to circular blobs with larger radii. The post-processing needs are significantly diminished, necessitating only confidence filtering and non-maximum suppression, in contrast on the CHT system, which requires computationally demanding masking, thresholding, and radius filtering. Collectively, these characteristics underscore the practical and clinical viability of YOLOv9 as a screening instrument that integrates speed, precision, and versatility.

Looking at individual cases gives more evidence that the system is consistent and reliable. For images with a lot of MA counts, like 45_right (true count = 63) and 58_right (true count = 48), YOLOv9 got the numbers very close to the true numbers (62 and 48, respectively), but the CHT-based system always got them wrong. In images with fewer lesions, like 53_left (true count = 12) and 59_left (true count = 5), YOLOv9 still found them correctly, but the CHT method sometimes over- or undercounted them. These findings indicate that the deep learning model is resilient across various lesion densities, a crucial factor for clinical screening where patient variability is anticipated.

When compared to previous studies, these results add to the growing body of evidence that convolutional neural networks (CNNs) can greatly improve the analysis of retinal images. Standard geometry-based methods, such as CHT, often face limitations stemming from rigid assumptions about lesion morphology and size. On the other hand, CNN-based methods directly pull out useful features from the data, which makes them flexible enough to work with different types of lesions. The findings of this study substantiate the hypothesis that deep learning can enhance both the sensitivity and specificity of automated microaneurysm detection, thereby facilitating earlier and more precise identification of diabetic retinopathy.

Unlike CHT, which relies on rigid geometric assumptions (for example perfect circularity of objects), YOLOv9 leverages a deep convolutional backbone with enhanced feature extraction, multi-scale detection heads, and anchor-free detection strategies. These design improvements allow YOLOv9 to detect objects of varying shapes, sizes, and orientations with high accuracy, overcoming the limitations of CHT’s rigid circularity assumptions. Moreover, the improved attention mechanisms and feature fusion layers in YOLOv9 contribute to better localisation and robustness in complex images.

These findings offer several research avenues for the future. Further validation on larger and more diverse datasets would be necessary to confirm that the YOLOv9 approach is generalisable across different populations and imaging technologies. Examining the integration of multimodal or temporal retinal data, such as optical coherence tomography and fundus imaging, may also help uncover subtle lesions or early-stage disease. To ensure that automated systems can effectively support ophthalmologist in early diagnosis and patient care, it will be crucial to explore techniques to enhance model interpretability and clinical integration. This will help translate these advancements into routine screening procedures.

This work illustrates that YOLOv9 significantly surpasses conventional CHT-based methods, exhibiting enhanced accuracy, expedited processing, and flexibility to various lesion morphologies. CNN-based systems like YOLOv9 can substantially improve diabetic retinopathy screening programs and boost patient outcomes through reliable and consistent detection of microaneurysms. In addition, the proposed models successfully tackle the common challenges of small sample sizes and class imbalance, which are frequent limitations in medical datasets.

Although YOLOv9 demonstrated strong performance in our experiments, several limitations warrant consideration. Our dataset comprised a stratified subset of 800 images drawn from larger collections and it did not entirely capture the variability prevalent in broader populations. The lack of external validation also limits our ability to confirm generalisability. In addition, we did not conduct a detailed failure or lesion-size sensitivity analysis, which is important given that microaneurysms are extremely small and can be difficult for YOLO models to detect. Our multi-step preprocessing pipeline, while helpful for detection performance, may not prove that practical in real clinical settings due to increased processing complexity and limited robustness to variability in real-world images.

Image quality and acquisition differences, such as camera type, illumination, and operator skill were not explored in depth and could have a meaningful impact on the performance outside controlled datasets. Finally, although YOLOv9 surpassed CHT in our study, recent research employing YOLOv8 and microaneurysm-specific models has demonstrated robust outcomes, and our findings should be considered within the broader scope of this literature [35,36,37]. Upcoming benchmarking initiatives incorporating these contemporary methodologies will facilitate the clarification of the unique advantages offered by YOLOv9 [5,39].

## 6. Conclusions

This study shows that using YOLOv9 for microaneurysm detection offers a clear step forward in improving early diabetic retinopathy screening. Compared with traditional geometry-based methods, the YOLOv9 model delivers higher accuracy, faster processing, and more reliable results, even when image quality varies. Its ability to recognise very small and irregular lesions makes it especially valuable for identifying early signs of diabetic retinopathy, which are often subtle and easily missed. These findings highlight the potential of modern deep learning models like YOLOv9 to make retinal screening more accurate, efficient, and accessible in real-world healthcare settings.

YOLOv9 was selected over earlier versions because it offers several key improvements that make it particularly effective for medical image analysis. The model is specifically optimised to detect minute details in images, like microaneurysms, and is more robust to image noise. It also performs consistently in less-than ideal conditions. Its updated architecture and more advanced feature extraction allow it to identify subtle patterns and textures that older YOLO versions might miss. With these advantages, YOLOv9 is not only more accurate but also more flexible for practical clinical use, offering a solid foundation for upcoming advancements in automated diabetic retinopathy screening and other medical imaging applications.

## Figures and Tables

**Table 3:** System performance analysis.

	Criterion/Metric	System I: CHT-Based Pipeline	System II: YOLOv9-Based Pipeline
Pre-processing	Detection Method	Circular Hough Transform (geometry-based)	CNN-based object detection (YOLOv9)
	Input Requirement	Preprocessed binary candidate map	Raw or preprocessed retinal image (RGB/tiles)
Image processing	Sensitivity (Recall)	0.65–0.72	0.83–0.88
	Specificity (Precision)	0.60–0.68	0.82–0.87
	F1-score	0.62–0.70	0.82–0.87
	Accuracy for 200 images [5% tolerance]	56%	91%
	Mean Average Precision (mAP)	45%	89.55%
Infrastructure	Computation Time	~2–5 s per image (CPU)	~50–200 ms per tile (GPU)
	Object Size Handling	Limited to circular blobs, radius 5–25 px	Detects small, irregular lesions down to ~3–5 px
	Post-processing Load	High (masking, thresholding, radius filtering)	Moderate (confidence filtering, NMS)

**Table 4:** Microaneurysms (MA) detection performance.

Image Name (True MA Count)	System I: Detected by CHT	System II: Detected by YOLOv9
45_right (63)	55	62
51_left (27)	32	27
52_right (41)	38	41
53_left (12)	15	12
54_right (8)	11	8
55_left (35)	30	34
56_right (22)	18	22
57_left (17)	20	17
58_right (48)	42	48
59_left (5)	4	5

## Data Availability

The data used to support the findings of this study are included within the article.
